# Alternative Entropy Measures and Generalized Khinchin–Shannon Inequalities

**DOI:** 10.3390/e23121618

**Published:** 2021-12-01

**Authors:** Rubem P. Mondaini, Simão C. de Albuquerque Neto

**Affiliations:** COPPE, Centre of Technology, Federal University of Rio de Janeiro, Rio de Janeiro 21941-901, Brazil; simao.albuquerqueneto@coppe.ufrj.br

**Keywords:** entropy measures, synergy, probabilistic distributions

## Abstract

The Khinchin–Shannon generalized inequalities for entropy measures in Information Theory, are a paradigm which can be used to test the Synergy of the distributions of probabilities of occurrence in physical systems. The rich algebraic structure associated with the introduction of escort probabilities seems to be essential for deriving these inequalities for the two-parameter Sharma–Mittal set of entropy measures. We also emphasize the derivation of these inequalities for the special cases of one-parameter Havrda–Charvat’s, Rényi’s and Landsberg–Vedral’s entropy measures.

## 1. Introduction

In the present contribution we derive the Generalized Khinchin–Shannon inequalities (GKS) [1,2] associated to entropy measures of the Sharma–Mittal (SM) set [3]. We stress that the derivations to be presented here are a tentative way of implementing the ideas of the literature on interdisciplinary topics of Statistical Mechanics and Theory of Information [4,5,6]. The algebraic structure of the escort probability distributions on these derivations seems to be essential, contrary to the intuitive derivation of the usual Khinchin–Shannon inequalities for the Gibbs–Shannon entropy measures. We start on Section 2 with the construction of a generic probabilistic space with their elements—the probabilities of occurrence—arranged on blocks of *m* rows and *n* columns. It then follows the introduction of the definitions of simple, joint, conditional and marginal probabilities through the use of the Bayes’ law. In Section 3, we make use of the assumption of concavity in order to unveil the Synergy of the distribution of values of Gibbs–Shannon entropy measures [2]. In Section 4, we present the same development but for the SM set of entropy measures, after the introduction of the concept of escort probabilities. We then specialize the derivations to Havrda–Charvat’s, Rényi’s and Landsberg–Vedral’s entropies [7,8,9]. A detailed study is then undertaken in this section to treat the eventual ordering between the probabilities of occurrence and their associated escort probabilities. This is then enough for deriving the GKS inequalities for the SM entropy measures. In Section 5, we present a proposal for Information measure associated to SM entropies and we derive its related inequalities [10]. At this point we stress once more the upsurge of the synergy effect on the comparison of the information obtained from the entropy calculated with joint probabilities of occurrence and the entropies corresponding to simple probabilities. In Section 6, we present an alternative derivation of the GKS inequalities based on Hölder inequalities [11]. These can provide, in association with Bayes’ law, the same assumptions of concavity which have been used in Section 3 and Section 4 and a consequent identical derivation of the GKS inequalities given in Section 4.

## 2. The Probability Space. Probabilities of Occurrence

We consider that the data could be represented on two-dimensional arrays of *m* rows and *n* columns. We then have m×n blocks of data to undertake the statistical analysis. The joint probabilities of occurrence of a set of *t* variables $a_{1},\ldots ,a_{t}$ in columns $j_{1},\ldots ,j_{t}$, respectively, are given by

(1) $$p_{j_{1}\ldots j_{t}}\left(a_{1},\ldots ,a_{t}\right)=\frac{n_{j_{1}\ldots j_{t}}\left(a_{1},\ldots ,a_{t}\right)}{m}\;,$$

where *m* is the number of rows in the subarray m×t of the array m×n, and $n_{j_{1}\ldots j_{t}}\left(a_{1},\ldots ,a_{t}\right)$ is the number of occurrences of the set $a_{1},\ldots ,a_{t}$. The values assumed by the variables $j_{1},\ldots ,j_{t}$, $j_{1}<j_{2}<\ldots <j_{t}$, are respectively given by:

(2) $$\begin{array}{cc} j_{1} & =1,2,\ldots ,n-t+1\\ j_{2} & =j_{1}+1,j_{1}+2,\ldots ,n-t+2\\ \vdots & \\ j_{t-1} & =j_{t-2}+1,j_{t-2}+2,\ldots ,n-1\\ j_{t} & =j_{t-1}+1,j_{t-1}+2,\ldots ,n\;, \end{array}$$

or,

(3) $$\begin{array}{cc} j_{1} & =1,2,\ldots ,n-t+1\\ j_{2} & =j_{1}+1,j_{1}+2,\ldots ,n-t+2\\ \vdots & \\ j_{t-1} & =j_{1}+t-2,j_{1}+t-1,\ldots ,n-1\\ j_{t} & =j_{1}+t-1,j_{1}+t,\ldots ,n\;. \end{array}$$

There are then nt=n!t!(n−t)! objects of *t* columns each, 1≤t≤n, and if the variables $a_{1},\ldots ,a_{t}$ take on values 1,…,W, then we will have ${\left(W\right)}^{t}$ components for each of these objects.

Since:

(4) $$\sum_{a_{1},\ldots ,a_{t}=1}^{W}n_{j_{1}\ldots j_{t}}\left(a_{1},\ldots ,a_{t}\right)=m\;,$$

we can write:

(5) $$\sum_{a_{1},\ldots ,a_{t}=1}^{W}p_{j_{1}\ldots j_{t}}\left(a_{1},\ldots ,a_{t}\right)=1\;.$$

On the study of distributions of bases of nucleotides or distributions of amino acids in proteins, the related values of *W* are W=4 and W=20, respectively.

The Bayes’ law for the probabilities of occurrence of Equation (1) is written as:

(6) $$\begin{array}{cc} p_{j_{1}\ldots j_{t}}\left(a_{1},\ldots ,a_{t}\right) & =p_{j_{1}\ldots j_{t}}\left(a_{1},\ldots ,a_{t-1}|a_{t}\right)\cdot p_{j_{t}}\left(a_{t}\right)\\ \; & =p_{j_{t}j_{1}\ldots j_{t-1}}\left(a_{t}|a_{1},\ldots ,a_{t-1}\right)\cdot p_{j_{1}\ldots j_{t-1}}\left(a_{1},\ldots ,a_{t-1}\right)\\ \; & =p_{j_{t}j_{1}\ldots j_{t-1}}\left(a_{t},a_{1},\ldots ,a_{t-1}\right)\;, \end{array}$$

where $p_{j_{1}\ldots j_{t}}\left(a_{1},\ldots ,a_{t-1}|a_{t}\right)$ stands for the conditional probability of occurrence of the values associated to the variables $a_{1},\ldots ,a_{t-1}$ in the columns $j_{1},\ldots ,j_{t-1}$, respectively, if the values associated to $a_{t}$ in the *j*th column are given a priori. This also means that:

(7) $$\sum_{a_{1},\ldots ,a_{t-1}}p_{j_{1}\ldots j_{t}}\left(a_{1},\ldots ,a_{t-1}|a_{t}\right)=1\;.$$

The marginal probabilities related to $p_{j_{1}\ldots j_{t}}\left(a_{1},\ldots ,a_{t}\right)$ are then given by

(8) $$\begin{array}{cc} p_{j_{t}}\left(a_{t}\right) & =\sum_{a_{1},\ldots ,a_{t-1}}p_{j_{1}\ldots j_{t}}\left(a_{1},\ldots ,a_{t}\right)\\ \; & =\sum_{a_{1},\ldots ,a_{t-1}}p_{j_{1}\ldots j_{t}}\left(a_{1},\ldots ,a_{t-1}|a_{t}\right)p_{j_{t}}\left(a_{t}\right)\;. \end{array}$$

We then have from Equations (6) and (8):

(9) $$1=\sum_{a_{t}}p_{j_{t}}\left(a_{t}\right)=\sum_{a_{1},\ldots ,a_{t}}p_{j_{1}\ldots j_{t}}\left(a_{1},\ldots ,a_{t}\right)\;,$$

which is the same result of Equation (5).

## 3. The Assumption of Concavity and the Synergy of Gibbs–Shannon Entropy Measures

A concave function of several variables should satisfy the following inequality:

(10) $$\sum_{l}\lambda_{l}f\left(x_{l}\right)\leq f\left(\sum_{l}\lambda_{l}x_{l}\right)\;;\;\lambda_{l}\geq 0\;;\;\sum_{l}\lambda_{l}=1\;.$$

We shall apply Equation (10) to the Gibbs–Shannon entropies:

(11) $$S_{j_{1}\ldots j_{t}}=-\sum_{a_{1},\ldots ,a_{t}}p_{j_{1}\ldots j_{t}}\left(a_{1},\ldots ,a_{t}\right)\log p_{j_{1}\ldots j_{t}}\left(a_{1},\ldots ,a_{t}\right)\;,$$

(12) $$S_{j_{1}\ldots j_{t-1}|j_{t}}=-\sum_{a_{1},\ldots ,a_{t}}p_{j_{t}}\left(a_{t}\right)p_{j_{1}\ldots j_{t}}\left(a_{1},\ldots ,a_{t-1}|a_{t}\right)\log p_{j_{1}\ldots j_{t}}\left(a_{1},\ldots ,a_{t-1}|a_{t}\right)\;.$$

where Equation (12) stands for the definition of Gibbs–Shannon entropy which is related to the conditional probabilities $p_{j_{1}\ldots j_{t}}\left(a_{1},\ldots ,a_{t-1}|a_{t}\right)$. It is a measure of the uncertainty [2] on the distribution of probabilities of the columns $j_{1},\ldots ,j_{t-1}$, when we have previous information on the distribution of the column $j_{t}$.

From Bayes’ law, Equation (6) and from Equations (8), (11) and (12), we get:

(13) $$S_{j_{1}\ldots j_{t}}=S_{j_{1}\ldots j_{t-1}|j_{t}}+S_{j_{t}}\;.$$

We now use the correspondences:

(14) $$\begin{array}{c} \lambda_{l}↔p_{j_{t}}\left(a_{t}\right)\;;\;x_{l}↔p_{j_{1}\ldots j_{t}}\left(a_{1},\ldots ,a_{t-1}|a_{t}\right)\;,\\ f\left(x_{l}\right)↔-\sum_{a_{1},\ldots ,a_{t-1}}p_{j_{1}\ldots j_{t}}\left(a_{1},\ldots ,a_{t-1}|a_{t}\right)\log p_{j_{1}\ldots j_{t}}\left(a_{1},\ldots ,a_{t-1}|a_{t}\right)\;, \end{array}$$

and we then have:

(15) $$\sum_{l}\lambda_{l}x_{l}↔\sum_{a_{t}}p_{j_{t}}\left(a_{t}\right)p_{j_{1}\ldots j_{t}}\left(a_{1},\ldots ,a_{t-1}|a_{t}\right)=\sum_{a_{t}}p_{j_{1}\ldots j_{t}}\left(a_{1},\ldots ,a_{t}\right)=p_{j_{1}\ldots j_{t-1}}\left(a_{1},\ldots ,a_{t-1}\right)\;,$$

(16) $$\sum_{l}\lambda_{l}f\left(x_{l}\right)↔-\sum_{a_{t},a_{1},\ldots ,a_{t-1}}p_{j_{t}}\left(a_{t}\right)p_{j_{1}\ldots j_{t}}\left(a_{1},\ldots ,a_{t-1}|a_{t}\right)\log p_{j_{1}\ldots j_{t}}\left(a_{1},\ldots ,a_{t-1}|a_{t}\right)\;,$$

and

(17) $$f\left(\sum_{l}\lambda_{l}x_{l}\right)↔-\sum_{a_{1},\ldots ,a_{t-1}}p_{j_{1}\ldots j_{t-1}}\left(a_{1},\ldots ,a_{t-1}\right)\log p_{j_{1}\ldots j_{t-1}}\left(a_{1},\ldots ,a_{t-1}\right)\;.$$

After substituting Equations (12), (16) and (17) into Equation (10), we get:

(18) $$\begin{array}{cc} S_{j_{1}\ldots j_{t-1}|j_{t}} & =-\sum_{a_{1},\ldots ,a_{t}}p_{j_{t}}\left(a_{t}\right)p_{j_{1}\ldots j_{t}}\left(a_{1},\ldots ,a_{t-1}|a_{t}\right)\log p_{j_{1}\ldots j_{t}}\left(a_{1},\ldots ,a_{t-1}|a_{t}\right)\\ \; & \leq -\sum_{a_{1},\ldots ,a_{t-1}}p_{j_{1}\ldots j_{t-1}}\left(a_{1},\ldots ,a_{t-1}\right)\log p_{j_{1}\ldots j_{t-1}}\left(a_{1},\ldots ,a_{t-1}\right)\;, \end{array}$$

or,

(19) $$S_{j_{1}\ldots j_{t-1}|j_{t}}\leq S_{j_{1}\ldots j_{t-1}}\;.$$

This means that the uncertainty of the distribution on the columns $j_{1},\ldots ,j_{t-1}$ cannot be increased when we have previous information on the distribution of column $j_{t}$.

From Equations (13) and (19), we then write:

(20) $$S_{j_{1}\ldots j_{t}}\leq S_{j_{1}\ldots j_{t-1}}+S_{j_{t}}\;,$$

and by iteration we get the Khinchin–Shannon inequality for the Gibbs–Shannon entropy measure:

(21) $$S_{j_{1}\ldots j_{t}}\leq \sum_{l=1}^{t}S_{j_{l}}\;.$$

The usual meaning given to Equation (21) is that the minimum of the information to be obtained from the analysis of the joint probabilities of a set of *t* columns is given by the sum of the informations associated with the *t* columns if considered as independent [1,2,10]. This is also seen as an aspect of Synergy [12,13] of the distribution of probabilities of occurrence.

## 4. The Assumption of Concavity and the Synergy of Sharma–Mittal (SM) Entropy Measures. The GKS Inequalities

We shall now use the assumption of concavity given by Equation (10) on Sharma–Mittal (SM) entropy measures:

(22) $${\left(SM\right)}_{j_{1}\ldots j_{t}}=\frac{{\left(\alpha_{j_{1}\ldots j_{t}}\right)}^{\frac{1-r}{1-s}}-1}{1-r}\;,$$

where,

(23) $$\alpha_{j_{1}\ldots j_{t}}=\sum_{a_{1},\ldots ,a_{t}}{\left[p_{j_{1}\ldots j_{t}}\left(a_{1},\ldots ,a_{t}\right)\right]}^{s}\;,$$

and *r*, *s* are non-dimensional parameters.

Analogously to Equation (12), we also introduce the “conditional entropy measure”

(24) $${\left(SM\right)}_{j_{1}\ldots j_{t-1}|j_{t}}=\frac{{\left(\beta_{j_{1}\ldots j_{t-1}|j_{t}}\right)}^{\frac{1-r}{1-s}}-1}{1-r}\;,$$

where

(25) $$\beta_{j_{1}\ldots j_{t-1}|j_{t}}=\sum_{a_{1},\ldots ,a_{t}}{\hat{p}}_{j_{t}}\left(a_{t}\right){\left[p_{j_{1}\ldots j_{t}}\left(a_{1},\ldots ,a_{t-1}|a_{t}\right)\right]}^{s}\;,$$

and ${\hat{p}}_{j_{t}}\left(a_{t}\right)$ stands for the escort probability

(26) $${\hat{p}}_{j_{t}}\left(a_{t}\right)=\frac{{\left(p_{j_{t}}\left(a_{t}\right)\right)}^{s}}{\alpha_{j_{t}}}=\frac{{\left(p_{j_{t}}\left(a_{t}\right)\right)}^{s}}{\sum_{a_{t}}{\left(p_{j_{t}}\left(a_{t}\right)\right)}^{s}}\;.$$

We have in general:

(27) $${\hat{p}}_{j_{1}\ldots j_{t}}\left(a_{1},\ldots ,a_{t}\right)=\frac{{\left(p_{j_{1}\ldots j_{t}}\left(a_{1},\ldots ,a_{t}\right)\right)}^{s}}{\alpha_{j_{1}\ldots j_{t}}}=\frac{{\left(p_{j_{1}\ldots j_{t}}\left(a_{1},\ldots ,a_{t}\right)\right)}^{s}}{\sum_{a_{1},\ldots ,a_{t}}{\left(p_{j_{1}\ldots j_{t}}\left(a_{1},\ldots ,a_{t}\right)\right)}^{s}}\;.$$

The inverse transformations are given by:

(28) $$p_{j_{t}}\left(a_{t}\right)=\frac{{\left({\hat{p}}_{j_{t}}\left(a_{t}\right)\right)}^{1/s}}{\sum_{a_{t}}{\left({\hat{p}}_{j_{t}}\left(a_{t}\right)\right)}^{1/s}}\;,$$

(29) $$p_{j_{1}\ldots j_{t}}\left(a_{1},\ldots ,a_{t}\right)=\frac{{\left({\hat{p}}_{j_{1}\ldots j_{t}}\left(a_{1},\ldots ,a_{t}\right)\right)}^{1/s}}{\sum_{a_{1},\ldots ,a_{t}}{\left({\hat{p}}_{j_{1}\ldots j_{t}}\left(a_{1},\ldots ,a_{t}\right)\right)}^{1/s}}\;,$$

with

(30) $$\sum_{a_{t}}p_{j_{t}}\left(a_{t}\right)=1=\sum_{a_{t}}{\hat{p}}_{j_{t}}\left(a_{t}\right)\;,$$

(31) $$\sum_{a_{1},\ldots ,a_{t}}p_{j_{1}\ldots j_{t}}\left(a_{1},\ldots ,a_{t}\right)=1=\sum_{a_{1},\ldots ,a_{t}}{\hat{p}}_{j_{1}\ldots j_{t}}\left(a_{1},\ldots ,a_{t}\right)\;.$$

A range of variation for the parameters *r*, *s* of Sharma–Mittal entropies, Equation (22), should be derived from a requirement for strict concavity. In order to do so, let us remember that for each set of *t* columns (m×t subarray) there are *m* rows of *t* values each (*t*-sequences). We now denote these *t*-sequences by:

(32) $$(a_{1}^{q_{\mu }},\ldots ,a_{t}^{q_{\mu }})\;,\;\mu =1,\ldots ,m$$

A sufficient requirement for strict concavity is the negative definiteness of the quadratic form associated to the Hessian matrix [14], whose elements are given by:

(33) $$H_{q_{\mu }q_{\nu }}=\frac{\partial^{2}{\left(SM\right)}_{j_{1}\ldots j_{t}}}{\partial p_{j_{1}\ldots j_{t}}\left(a_{1}^{q_{\mu }},\ldots ,a_{t}^{q_{\mu }}\right)\partial p_{j_{1}\ldots j_{t}}\left(a_{1}^{q_{\nu }},\ldots ,a_{t}^{q_{\nu }}\right)}\;,\;\mu ,\nu =1,\ldots ,m\;.$$

We then consider the *m* submatrices along the diagonal of the Hessian matrix. Their determinants should be alternately negative or positive according to whether their order is odd or even [15], respectively:

(34) $$\det H_{q_{\mu }q_{\nu }}\left(\mu ,\nu =1\right)=s{\left(\alpha_{j_{1}\ldots j_{t}}\right)}^{\frac{s-r}{1-s}}{\left[p_{j_{1}\ldots j_{t}}\left(a_{1}^{q_{1}},\ldots ,a_{t}^{q_{1}}\right)\right]}^{s-2}\left[\frac{s(s-r)}{{\left(1-s\right)}^{2}}\cdot {\hat{p}}_{j_{1}\ldots j_{t}}\left(a_{1}^{q_{1}},\ldots ,a_{t}^{q_{1}}\right)-1\right]\;,$$

(35) $$\begin{array}{cc} \det H_{q_{\mu }q_{\nu }}\left(\mu ,\nu =1,2\right)=-s^{2}{\left(\alpha_{j_{1}\ldots j_{t}}\right)}^{2\left(\frac{s-r}{1-s}\right)}{\left[p_{j_{1}\ldots j_{t}}\left(a_{1}^{q_{1}},\ldots ,a_{t}^{q_{1}}\right)\cdot p_{j_{1}\ldots j_{t}}\left(a_{1}^{q_{2}},\ldots ,a_{t}^{q_{2}}\right)\right]}^{s-2}\; & \\ \left\{\frac{s(s-r)}{{\left(1-s\right)}^{2}}\cdot \left[{\hat{p}}_{j_{1}\ldots j_{t}}\left(a_{1}^{q_{1}},\ldots ,a_{t}^{q_{1}}\right)+{\hat{p}}_{j_{1}\ldots j_{t}}\left(a_{1}^{q_{2}},\ldots ,a_{t}^{q_{2}}\right)\right]-1\right\}\;, & \end{array}$$

(36) $$\begin{array}{cc} \det H_{q_{\mu }q_{\nu }}\left(\mu ,\nu =1,2,3\right)=s^{3}{\left(\alpha_{j_{1}\ldots j_{t}}\right)}^{3\left(\frac{s-r}{1-s}\right)}{\left[p_{j_{1}\ldots j_{t}}\left(a_{1}^{q_{1}},\ldots ,a_{t}^{q_{1}}\right)\cdot p_{j_{1}\ldots j_{t}}\left(a_{1}^{q_{2}},\ldots ,a_{t}^{q_{2}}\right)\cdot p_{j_{1}\ldots j_{t}}\left(a_{1}^{q_{3}},\ldots ,a_{t}^{q_{3}}\right)\right]}^{s-2} & \\ \cdot \left\{\frac{s(s-r)}{{\left(1-s\right)}^{2}}\cdot \left[{\hat{p}}_{j_{1}\ldots j_{t}}\left(a_{1}^{q_{1}},\ldots ,a_{t}^{q_{1}}\right)+{\hat{p}}_{j_{1}\ldots j_{t}}\left(a_{1}^{q_{2}},\ldots ,a_{t}^{q_{2}}\right)+{\hat{p}}_{j_{1}\ldots j_{t}}\left(a_{1}^{q_{3}},\ldots ,a_{t}^{q_{3}}\right)\right]-1\right\}\;. & \end{array}$$

We then choose:

(37) 1>r≥s>0,

and we have from Equations (34)–(36):

(38) $$\det H_{q_{\mu }q_{\nu }}\left(\mu ,\nu =1\right)<0\;,$$

(39) $$\det H_{q_{\mu }q_{\nu }}\left(\mu ,\nu =1,2\right)>0\;,$$

(40) $$\det H_{q_{\mu }q_{\nu }}\left(\mu ,\nu =1,2,3\right)<0\;.$$

We then have generally:

(41) $$\begin{array}{cc} \det H_{q_{\mu }q_{\nu }}\left(\mu ,\nu =1,\ldots ,m\right)={\left(-1\right)}^{m-1}\cdot s^{m}{\left(\alpha_{j_{1}\ldots j_{t}}\right)}^{m\left(\frac{s-r}{1-s}\right)}{\left[\prod_{\mu =1}^{m}p_{j_{1}\ldots j_{t}}\left(a_{1}^{q_{\mu }},\ldots ,a_{t}^{q_{\mu }}\right)\right]}^{s-2} & \\ \left\{\frac{s(s-r)}{{\left(1-s\right)}^{2}}\sum_{\mu =1}^{m}{\hat{p}}_{j_{1}\ldots j_{t}}\left(a_{1}^{q_{\mu }},\ldots ,a_{t}^{q_{\mu }}\right)-1\right\}\;. \end{array}$$

This completes the proof.

From Bayes’ law, Equation (6) and from Equations (8), (22)–(25), we can write:

(42) $${\left(SM\right)}_{j_{1}\ldots j_{t}}={\left(SM\right)}_{j_{1}\ldots j_{t-1}|j_{t}}+{\left(SM\right)}_{j_{t}}+\left(1-r\right){\left(SM\right)}_{j_{1}\ldots j_{t-1}|j_{t}}\cdot {\left(SM\right)}_{j_{t}}\;.$$

We are now ready to use the concavity assumption, Equation (10) for deriving the GKS inequalities. In order to do so, we make the correspondences:

(43) $$\begin{array}{c} \lambda_{l}↔{\hat{p}}_{j_{t}}\left(a_{t}\right)\;;\;x_{l}↔p_{j_{1}\ldots j_{t}}\left(a_{1},\ldots ,a_{t-1}|a_{t}\right)\;,\\ f\left(x_{l}\right)↔\sum_{a_{1},\ldots ,a_{t-1}}{\left[p_{j_{1}\ldots j_{t}}\left(a_{1},\ldots ,a_{t-1}|a_{t}\right)\right]}^{s}\;. \end{array}$$

We can then write:

(44) $$\sum_{l}\lambda_{l}x_{l}↔\sum_{a_{t}}{\hat{p}}_{j_{t}}\left(a_{t}\right)p_{j_{1}\ldots j_{t}}\left(a_{1},\ldots ,a_{t-1}|a_{t}\right)\;,$$

(45) $$\sum_{l}\lambda_{l}f\left(x_{l}\right)↔\sum_{a_{t},a_{1},\ldots ,a_{t-1}}{\hat{p}}_{j_{t}}\left(a_{t}\right){\left[p_{j_{1}\ldots j_{t}}\left(a_{1},\ldots ,a_{t-1}|a_{t}\right)\right]}^{s}\;,$$

and

(46) $$f\left(\sum_{l}\lambda_{l}x_{l}\right)↔\sum_{a_{1},\ldots ,a_{t-1}}{\left[\sum_{a_{t}}{\hat{p}}_{j_{t}}\left(a_{t}\right)p_{j_{1}\ldots j_{t}}\left(a_{1},\ldots ,a_{t-1}|a_{t}\right)\right]}^{s}\;.$$

With the correspondences above, Equation (10) will turn into:

(47) $$\sum_{a_{1},\ldots ,a_{t-1}}{\left[\sum_{a_{t}}{\hat{p}}_{j_{t}}\left(a_{t}\right)p_{j_{1}\ldots j_{t}}\left(a_{1},\ldots ,a_{t-1}|a_{t}\right)\right]}^{s}\geq \sum_{a_{1},\ldots ,a_{t}}{\hat{p}}_{j_{t}}\left(a_{t}\right){\left[p_{j_{1}\ldots j_{t}}\left(a_{1},\ldots ,a_{t-1}|a_{t}\right)\right]}^{s}\;.$$

An additional information should be taken into consideration before we derive the GKS inequalities:

On each $j_{t}$ column, t=1,…,n of a m×n block, there will be values $a_{t}^{\prime }$, $a_{t}^{\prime \prime }$ of $a_{t}$ such that

(48) $$p_{j_{t}}\left(a_{t}^{\prime }\right)\geq {\hat{p}}_{j_{t}}\left(a_{t}^{\prime }\right)\;,$$

and

(49) $$p_{j_{t}}\left(a_{t}^{\prime \prime }\right)\leq {\hat{p}}_{j_{t}}\left(a_{t}^{\prime \prime }\right)\;.$$

After multiplying inequalities (48) and (49) by $p_{j_{1}\ldots j_{t}}\left(a_{1},\ldots ,a_{t-1}|a_{t}^{\prime }\right)$ and $p_{j_{1}\ldots j_{t}}\left(a_{1},\ldots ,a_{t-1}|a_{t}^{\prime \prime }\right)$, respectively, and summing up in $a_{t}^{\prime }$ and $a_{t}^{\prime \prime }$, respectively, we get:

(50) $$\sum_{a_{t}^{\prime }}p_{j_{t}}\left(a_{t}^{\prime }\right)p_{j_{1}\ldots j_{t}}\left(a_{1},\ldots ,a_{t-1}|a_{t}^{\prime }\right)\geq \sum_{a_{t}^{\prime }}{\hat{p}}_{j_{t}}\left(a_{t}^{\prime }\right)p_{j_{1}\ldots j_{t}}\left(a_{1},\ldots ,a_{t-1}|a_{t}^{\prime }\right)\;,$$

and

(51) $$\sum_{a_{t}^{\prime \prime }}p_{j_{t}}\left(a_{t}^{\prime \prime }\right)p_{j_{1}\ldots j_{t}}\left(a_{1},\ldots ,a_{t-1}|a_{t}^{\prime \prime }\right)\leq \sum_{a_{t}^{\prime \prime }}{\hat{p}}_{j_{t}}\left(a_{t}^{\prime \prime }\right)p_{j_{1}\ldots j_{t}}\left(a_{1},\ldots ,a_{t-1}|a_{t}^{\prime \prime }\right)\;.$$

From Equations (48) and (49), any sum over the $a_{t}$ values can be partitioned as sums over the sets of values $a_{t}^{\prime }$ and $a_{t}^{\prime \prime }$:

(52) $$\sum_{a_{t}}p_{j_{t}}\left(a_{t}\right)p_{j_{1}\ldots j_{t}}\left(a_{1},\ldots ,a_{t-1}|a_{t}\right)=\sum_{a_{t}^{\prime }}p_{j_{t}}\left(a_{t}^{\prime }\right)p_{j_{1}\ldots j_{t}}\left(a_{1},\ldots ,a_{t-1}|a_{t}^{\prime }\right)+\sum_{a_{t}^{\prime \prime }}p_{j_{t}}\left(a_{t}^{\prime \prime }\right)p_{j_{1}\ldots j_{t}}\left(a_{1},\ldots ,a_{t-1}|a_{t}^{\prime \prime }\right)\;.$$

Substituting Equation (52) into Equations (50) and (51), we have:

(53) $$\begin{array}{cc} \sum_{a_{t}} & p_{j_{t}}\left(a_{t}\right)p_{j_{1}\ldots j_{t}}\left(a_{1},\ldots ,a_{t-1}|a_{t}\right)-\sum_{a_{t}^{\prime \prime }}p_{j_{t}}\left(a_{t}^{\prime \prime }\right)p_{j_{1}\ldots j_{t}}\left(a_{1},\ldots ,a_{t-1}|a_{t}^{\prime \prime }\right)\\ \; & \geq \sum_{a_{t}}{\hat{p}}_{j_{t}}\left(a_{t}\right)p_{j_{1}\ldots j_{t}}\left(a_{1},\ldots ,a_{t-1}|a_{t}\right)-\sum_{a_{t}^{\prime \prime }}{\hat{p}}_{j_{t}}\left(a_{t}^{\prime \prime }\right)p_{j_{1}\ldots j_{t}}\left(a_{1},\ldots ,a_{t-1}|a_{t}^{\prime \prime }\right)\;, \end{array}$$

and

(54) $$\begin{array}{cc} \sum_{a_{t}} & p_{j_{t}}\left(a_{t}\right)p_{j_{1}\ldots j_{t}}\left(a_{1},\ldots ,a_{t-1}|a_{t}\right)-\sum_{a_{t}^{\prime }}p_{j_{t}}\left(a_{t}^{\prime }\right)p_{j_{1}\ldots j_{t}}\left(a_{1},\ldots ,a_{t-1}|a_{t}^{\prime }\right)\\ \; & \leq \sum_{a_{t}}{\hat{p}}_{j_{t}}\left(a_{t}\right)p_{j_{1}\ldots j_{t}}\left(a_{1},\ldots ,a_{t-1}|a_{t}\right)-\sum_{a_{t}^{\prime }}{\hat{p}}_{j_{t}}\left(a_{t}^{\prime }\right)p_{j_{1}\ldots j_{t}}\left(a_{1},\ldots ,a_{t-1}|a_{t}^{\prime }\right)\;, \end{array}$$

respectively.

After applying the Bayes’ law, Equation (6), to the first term on the left hand side of Equations (53) and (54), we get:

(55) $$p_{j_{1}\ldots j_{t-1}}\left(a_{1},\ldots ,a_{t-1}\right)\geq \sum_{a_{t}}{\hat{p}}_{j_{t}}\left(a_{t}\right)p_{j_{1}\ldots j_{t}}\left(a_{1},\ldots ,a_{t-1}|a_{t}\right)+B^{\prime \prime }\;,$$

(56) $$p_{j_{1}\ldots j_{t-1}}\left(a_{1},\ldots ,a_{t-1}\right)\leq \sum_{a_{t}}{\hat{p}}_{j_{t}}\left(a_{t}\right)p_{j_{1}\ldots j_{t}}\left(a_{1},\ldots ,a_{t-1}|a_{t}\right)+B^{\prime }\;,$$

where

(57) $$B^{\prime \prime }=\sum_{a_{t}^{\prime \prime }}\left(p_{j_{t}}\left(a_{t}^{\prime \prime }\right)-{\hat{p}}_{j_{t}}\left(a_{t}^{\prime \prime }\right)\right)\cdot p_{j_{1}\ldots j_{t}}\left(a_{1},\ldots ,a_{t-1}|a_{t}^{\prime \prime }\right)\;,$$

(58) $$B^{\prime }=\sum_{a_{t}^{\prime }}\left(p_{j_{t}}\left(a_{t}^{\prime }\right)-{\hat{p}}_{j_{t}}\left(a_{t}^{\prime }\right)\right)\cdot p_{j_{1}\ldots j_{t}}\left(a_{1},\ldots ,a_{t-1}|a_{t}^{\prime }\right)\;,$$

and we have, $B^{\prime \prime }\leq 0$, $B^{\prime }\geq 0$, according to Equations (48) and (49), respectively.

After taking the *s*-power in Equations (55) and (56) and summing up in $a_{1},\ldots ,a_{t-1}$, we have:

(59) $$\sum_{a_{1},\ldots ,a_{t-1}}{\left[p_{j_{1}\ldots j_{t-1}}\left(a_{1},\ldots ,a_{t-1}\right)\right]}^{s}\geq \sum_{a_{1},\ldots ,a_{t-1}}{\left[\sum_{a_{t}}{\hat{p}}_{j_{t}}\left(a_{t}\right)p_{j_{1}\ldots j_{t}}\left(a_{1},\ldots ,a_{t-1}|a_{t}\right)+B^{\prime \prime }\right]}^{s}\;,$$

(60) $$\sum_{a_{1},\ldots ,a_{t-1}}{\left[p_{j_{1}\ldots j_{t-1}}\left(a_{1},\ldots ,a_{t-1}\right)\right]}^{s}\leq \sum_{a_{1},\ldots ,a_{t-1}}{\left[\sum_{a_{t}}{\hat{p}}_{j_{t}}\left(a_{t}\right)p_{j_{1}\ldots j_{t}}\left(a_{1},\ldots ,a_{t-1}|a_{t}\right)+B^{\prime }\right]}^{s}\;.$$

We now write, the concavity assumption, Equation (10), as:

(61) C≥D,

where

(62) $$C\equiv \sum_{a_{1},\ldots ,a_{t-1}}{\left[\sum_{a_{t}}{\hat{p}}_{j_{t}}\left(a_{t}\right)p_{j_{1}\ldots j_{t}}\left(a_{1},\ldots ,a_{t-1}|a_{t}\right)\right]}^{s}\;,$$

and

(63) $$D\equiv \sum_{a_{1},\ldots ,a_{t}}{\hat{p}}_{j_{t}}\left(a_{t}\right){\left[p_{j_{1}\ldots j_{t}}\left(a_{1},\ldots ,a_{t-1}|a_{t}\right)\right]}^{s}\;.$$

Equations (59) and (60) are now written as:

(64) $$A\geq C^{\prime \prime }\;,$$

(65) $$A\leq C^{\prime }\;,$$

where

(66) $$A\equiv \sum_{a_{1},\ldots ,a_{t-1}}{\left[p_{j_{1}\ldots j_{t-1}}\left(a_{1},\ldots ,a_{t-1}\right)\right]}^{s}\;,$$

and

(67) $$C^{\prime \prime }\equiv \sum_{a_{1},\ldots ,a_{t-1}}{\left[\sum_{a_{t}}{\hat{p}}_{j_{t}}\left(a_{t}\right)p_{j_{1}\ldots j_{t}}\left(a_{1},\ldots ,a_{t-1}|a_{t}\right)+B^{\prime \prime }\right]}^{s}\;,$$

(68) $$C^{\prime }\equiv \sum_{a_{1},\ldots ,a_{t-1}}{\left[\sum_{a_{t}}{\hat{p}}_{j_{t}}\left(a_{t}\right)p_{j_{1}\ldots j_{t}}\left(a_{1},\ldots ,a_{t-1}|a_{t}\right)+B^{\prime }\right]}^{s}\;.$$

Since $B^{\prime \prime }\leq 0$ and $B^{\prime }\geq 0$, we have trivially that:

(69) $$C^{\prime \prime }\leq C\;,$$

and

(70) $$C^{\prime }\geq C\;.$$

The set of inequalities, Equations (61), (64) and (69), or

(71) $$C\geq D\;;\;A\geq C^{\prime \prime }\;;\;C^{\prime \prime }\leq C\;,$$

and the set of inequalities, Equations (61), (65) and (70), or

(72) $$C\geq D\;;\;A\leq C^{\prime }\;;\;C^{\prime }\geq C\;,$$

can be arranged as the chains of inequalities

(73) $$C\geq A\geq D\geq C^{\prime \prime }\;,$$

and

(74) $$C^{\prime }\geq A\geq C\geq D\;,$$

respectively.

The inequality A≥D is common to the two chains above and it can be written as:

(75) $$\sum_{a_{1},\ldots ,a_{t-1}}{\left[p_{j_{1}\ldots j_{t-1}}\left(a_{1},\ldots ,a_{t-1}\right)\right]}^{s}\geq \sum_{a_{1},\ldots ,a_{t}}{\hat{p}}_{j_{t}}\left(a_{t}\right){\left[p_{j_{1}\ldots j_{t}}\left(a_{1},\ldots ,a_{t-1}|a_{t}\right)\right]}^{s}\;.$$

From the definition of the escort probabilities, Equations (26) and (27), we can write the right-hand side of Equation (75), as:

(76) $$\sum_{a_{1},\ldots ,a_{t}}{\hat{p}}_{j_{t}}\left(a_{t}\right){\left[p_{j_{1}\ldots j_{t}}\left(a_{1},\ldots ,a_{t-1}|a_{t}\right)\right]}^{s}=\frac{\sum_{a_{1},\ldots ,a_{t}}{\left[p_{j_{1}\ldots j_{t}}\left(a_{1},\ldots ,a_{t}\right)\right]}^{s}}{\sum_{a_{t}}{\left[p_{j_{t}}\left(a_{t}\right)\right]}^{s}}\;.$$

From Equations (75) and (76) and the definition of the α-symbols, Equation (23), we have:

(77) $$\alpha_{j_{1}\ldots j_{t}}\leq \alpha_{j_{1}\ldots j_{t-1}}\cdot \alpha_{j_{t}}\;.$$

We then get by iteration,

(78) $$\alpha_{j_{1}\ldots j_{t}}\leq \prod_{l=1}^{t}\alpha_{j_{l}}\;.$$

Equation (78) do correspond to the Generalized Khinchin–Shannon Inequalities (GKS) here derived for Sharma–Mittal entropies.

From Equations (22) and (37) we can also write for the GKS inequalities:

(79) $${\left(SM\right)}_{j_{1}\ldots j_{t}}\leq \frac{\prod_{l=1}^{t}\left[1+\left(1-r\right){\left(SM\right)}_{j_{l}}\right]-1}{1-r}\;.$$

The same words which have been written after Equation (21), could be written also here for the Sharma–Mittal entropy measures as the aspect of Synergy is concerned. We will introduce a proposal for information measure to stress this aspect on the next section.

For t=2,3, we can write from Equation (79):

(80) $${\left(SM\right)}_{j_{1}j_{2}}\leq {\left(SM\right)}_{j_{1}}+{\left(SM\right)}_{j_{2}}+\left(1-r\right){\left(SM\right)}_{j_{1}}{\left(SM\right)}_{j_{2}}\;,$$

(81) $$\begin{array}{cc} {\left(SM\right)}_{j_{1}j_{2}j_{3}}\leq {\left(SM\right)}_{j_{1}} & +{\left(SM\right)}_{j_{2}}+{\left(SM\right)}_{j_{3}}+\left(1-r\right)[{\left(SM\right)}_{j_{1}}{\left(SM\right)}_{j_{2}}+{\left(SM\right)}_{j_{2}}{\left(SM\right)}_{j_{3}}\\ \; & +{\left(SM\right)}_{j_{1}}{\left(SM\right)}_{j_{3}}]+{\left(1-r\right)}^{2}{\left(SM\right)}_{j_{1}}{\left(SM\right)}_{j_{2}}{\left(SM\right)}_{j_{3}}\;. \end{array}$$

The Havrda–Charvat’s, Rényi’s and Landsberg–Vedral’s entropies are easily obtained by taking the convenient limits in Equation (24):

(82) $$\lim_{r\to s}{\left(SM\right)}_{j_{1}\ldots j_{t}}={\left(HC\right)}_{j_{1}\ldots j_{t}}=\frac{\alpha_{j_{1}\ldots j_{t}}-1}{1-s}\;,$$

(83) $$\lim_{r\to 1}{\left(SM\right)}_{j_{1}\ldots j_{t}}=R_{j_{1}\ldots j_{t}}=\frac{\log(\alpha_{j_{1}\ldots j_{t}})}{1-s}\;,$$

(84) $$\lim_{r\to 2-s}{\left(SM\right)}_{j_{1}\ldots j_{t}}={\left(LV\right)}_{j_{1}\ldots j_{t}}=\frac{\alpha_{j_{1}\ldots j_{t}}-1}{\left(1-s\right)\alpha_{j_{1}\ldots j_{t}}}\;.$$

The Gibbs–Shannon entropy measure $S_{j_{1}\ldots j_{t}}$, Equation (11), is included in all these entropies through:

(85) $$\lim_{s\to 1}{\left(HC\right)}_{j_{1}\ldots j_{t}}=\lim_{s\to 1}R_{j_{1}\ldots j_{t}}=\lim_{s\to 1}{\left(LV\right)}_{j_{1}\ldots j_{t}}=S_{j_{1}\ldots j_{t}}\;.$$

Equations (83) and (85) have been derived via the l’Hôpital theorem.

For t=2,3, we write from Equations (82)–(84):

(86) $${\left(HC\right)}_{j_{1}j_{2}}\leq {\left(HC\right)}_{j_{1}}+{\left(HC\right)}_{j_{2}}+\left(1-s\right){\left(HC\right)}_{j_{1}}{\left(HC\right)}_{j_{2}}\;,$$

(87) $$\begin{array}{cc} {\left(HC\right)}_{j_{1}j_{2}j_{3}}\leq {\left(HC\right)}_{j_{1}} & +{\left(HC\right)}_{j_{2}}+{\left(HC\right)}_{j_{3}}+\left(1-s\right)[{\left(HC\right)}_{j_{1}}{\left(HC\right)}_{j_{2}}+{\left(HC\right)}_{j_{2}}{\left(HC\right)}_{j_{3}}\\ \; & +{\left(HC\right)}_{j_{1}}{\left(HC\right)}_{j_{3}}]+{\left(1-s\right)}^{2}{\left(HC\right)}_{j_{1}}{\left(HC\right)}_{j_{2}}{\left(HC\right)}_{j_{3}}\;, \end{array}$$

(88) $$R_{j_{1}j_{2}}\leq R_{j_{1}}+R_{j_{2}}\;,$$

(89) $$R_{j_{1}j_{2}j_{3}}\leq R_{j_{1}}+R_{j_{2}}+R_{j_{3}}\;,$$

(90) $${\left(LV\right)}_{j_{1}j_{2}}\leq {\left(LV\right)}_{j_{1}}+{\left(LV\right)}_{j_{2}}-\left(1-s\right){\left(LV\right)}_{j_{1}}{\left(LV\right)}_{j_{2}}\;,$$

(91) $$\begin{array}{cc} {\left(LV\right)}_{j_{1}j_{2}j_{3}}\leq {\left(LV\right)}_{j_{1}} & +{\left(LV\right)}_{j_{2}}+{\left(LV\right)}_{j_{3}}-\left(1-s\right)[{\left(LV\right)}_{j_{1}}{\left(LV\right)}_{j_{2}}+{\left(LV\right)}_{j_{2}}{\left(LV\right)}_{j_{3}}\\ \; & +{\left(LV\right)}_{j_{1}}{\left(LV\right)}_{j_{3}}]+{\left(1-s\right)}^{2}{\left(LV\right)}_{j_{1}}{\left(LV\right)}_{j_{2}}{\left(LV\right)}_{j_{3}}\;. \end{array}$$

As a last result of this section, we note that Equation (79) could be also derived from Equation (75), since this equation could be also written as:

(92) $${\left(SM\right)}_{j_{1}\ldots j_{t-1}|j_{t}}\leq {\left(SM\right)}_{j_{1}\ldots j_{t-1}}\;,$$

where we have used Equations (22)–(25).

After the comparison of Equation (42) and (92), we get the result of Equation (79) again.

## 5. An Information Measure Proposal Associated to Sharma–Mittal Entropy Measures

We are looking for a proposal of information measure which can fulfill a requirement of clear interpretation of the upsurge of Synergy in a probabilistic distribution and is supported by the usual idea of entropy as a measure of uncertainty.

For the Sharma–Mittal set of entropy measures the proposal for the associated information measure would be:

(93) $$I_{j_{1}\ldots j_{t}}=-\frac{{\left(SM\right)}_{j_{1}\ldots j_{t}}}{{\left(\alpha_{j_{1}\ldots j_{t}}\right)}^{\frac{1-r}{1-s}}}\;,\;\;1>r\geq s>0\;,$$

where ${\left(SM\right)}_{j_{1}\ldots j_{t}}$ and $\alpha_{j_{1}\ldots j_{t}}$ are given by Equations (22) and (23). We then have from Equation (93)

(94) $${\left(SM\right)}_{j_{1}\ldots j_{t}}=-\frac{I_{j_{1}\ldots j_{t}}}{1+\left(1-r\right)I_{j_{1}\ldots j_{t}}}\;.$$

From the GKS inequalities, Equation (79), and from Equation (94), we get:

(95) $$I_{j_{1}\ldots j_{t}}\geq \frac{\prod_{l=1}^{t}\left[1+\left(1-r\right)I_{j_{l}}\right]-1}{1-r}\geq \sum_{l=1}^{t}I_{j_{l}}\;.$$

The meaning of Equation (95) is that the minimum of information associated with *t* columns of probabilities of occurrence is given by the sum of informations associated to each column. This corresponds to the expression of Synergy of the distribution of probabilities of occurrences which we have derived on the previous section.

The inequalities (95), for t=2,3 are written as:

(96) $$I_{j_{1}j_{2}}\geq I_{j_{1}}+I_{j_{2}}+\left(1-r\right)I_{j_{1}}I_{j_{2}}\geq I_{j_{1}}+I_{j_{2}}\;,$$

(97) $$\begin{array}{cc} I_{j_{1}j_{2}j_{3}} & \geq I_{j_{1}}+I_{j_{2}}+I_{j_{3}}+\left(1-r\right)\left[I_{j_{1}}I_{j_{2}}+I_{j_{2}}I_{j_{3}}+I_{j_{1}}I_{j_{3}}\right]+{\left(1-r\right)}^{2}I_{j_{1}}I_{j_{2}}I_{j_{3}}\\ \; & \geq I_{j_{1}}+I_{j_{2}}+I_{j_{3}}\;. \end{array}$$

It seems worthwhile to derive yet another result which unveils once more the fundamental aspect of synergy of the distribution of probabilities of occurrence. From Equation (93), we have:

(98) $$dI_{j_{1}\ldots j_{t}}=-\frac{d{\left(SM\right)}_{j_{1}\ldots j_{t}}}{{\left(\alpha_{j_{1}\ldots j_{t}}\right)}^{2\left(\frac{1-r}{1-s}\right)}}\;,$$

and we then write from the GKS inequalities, Equation (78):

(99) $$\frac{1}{{\left(\alpha_{j_{1}\ldots j_{t}}\right)}^{2\left(\frac{1-r}{1-s}\right)}}\geq \frac{1}{\prod_{l=1}^{t}{\left(\alpha_{j_{l}}\right)}^{2\left(\frac{1-r}{1-s}\right)}}=\prod_{l=1}^{t}\frac{1}{{\left(\alpha_{j_{l}}\right)}^{2\left(\frac{1-r}{1-s}\right)}}\;.$$

We then get:

(100) $$-\frac{dI_{j_{1}\ldots j_{t}}}{d{\left(SM\right)}_{j_{1}\ldots j_{t}}}\geq \prod_{l=1}^{t}\left(-\frac{dI_{j_{l}}}{d{\left(SM\right)}_{j_{l}}}\right)\;.$$

Equation (100) do correspond to another result which originates from the Synergy of the distribution of probabilities of occurrence. It can be written as: The minimum of the rate of information increase with decreasing entropy in probability distribution for sets of *t* columns, is given by the product of the rates of information increase pertaining to each of the *t* columns.

## 6. The Use of Hölder’s Inequality for an Alternative Derivation of the GKS Inequalities

We firstly note that:

(101) $$\sum_{a_{t}}{\hat{p}}_{j_{t}}\left(a_{t}\right)p_{j_{1}\ldots j_{t}}\left(a_{1},\ldots ,a_{t-1}|a_{t}\right)=\frac{\sum_{a_{t}}p_{j_{1}\ldots j_{t}}\left(a_{1},\ldots ,a_{t}\right){\left(p_{j_{t}}\left(a_{t}\right)\right)}^{s-1}}{\sum_{a_{t}}{\left(p_{j_{t}}\left(a_{t}\right)\right)}^{s}}\;,$$

and we now introduce the Hölder’s inequality: Ref. [11]

(102) $${\left(\sum_{l}x_{l}y_{l}\right)}^{ab}\leq {\left(\sum_{l}{\left(x_{l}\right)}^{a}\right)}^{b}\cdot {\left(\sum_{l}{\left(y_{l}\right)}^{b}\right)}^{a}\;,\;\;ab=a+b\;,\;\;a\neq 1\;.$$

We can also write:

(103) $$\sum_{l}x_{l}y_{l}\geq {\left(\sum_{l}{\left(x_{l}\right)}^{a}\right)}^{\frac{1}{a}}\cdot {\left(\sum_{l}{\left(y_{l}\right)}^{b}\right)}^{\frac{1}{b}}\;,\;\;0\leq a<1\;,\;\;ab\leq 0\;,$$

or

(104) $${\left(\sum_{l}x_{l}y_{l}\right)}^{a}\geq \sum_{l}{\left(x_{l}\right)}^{a}\cdot {\left(\sum_{l}{\left(y_{l}\right)}^{b}\right)}^{\frac{a}{b}}\;,\;\;0\leq a<1\;,\;\;b\leq 0\;.$$

We now make the correspondences:

(105) $$x_{l}↔p_{j_{1}\ldots j_{t}}\left(a_{1},\ldots ,a_{t}\right)\;;\;y_{l}↔{\left(p_{j_{t}}\left(a_{t}\right)\right)}^{s-1}\;.$$

We take the *s*-power of the sides of Equation (101) and after using Equations (104) and (105) with a=s and $b=\frac{s}{s-1}$, we get:

(106) $$\begin{array}{cc} {\left(\sum_{a_{t}}{\hat{p}}_{j_{t}}\left(a_{t}\right)p_{j_{1}\ldots j_{t}}\left(a_{1},\ldots ,a_{t-1}|a_{t}\right)\right)}^{s} & ={\left(\frac{\sum_{a_{t}}p_{j_{1}\ldots j_{t}}\left(a_{1},\ldots ,a_{t}\right){\left(p_{j_{t}}\left(a_{t}\right)\right)}^{s-1}}{\sum_{a_{t}}{\left(p_{j_{t}}\left(a_{t}\right)\right)}^{s}}\right)}^{s}\\ \; & \geq \frac{\left(\sum_{a_{t}}{\left(p_{j_{1}\ldots j_{t}}\left(a_{1},\ldots ,a_{t}\right)\right)}^{s}\right){\left(\sum_{a_{t}}{\left(p_{j_{t}}\left(a_{t}\right)\right)}^{s}\right)}^{s-1}}{{\left(\sum_{a_{t}}{\left(p_{j_{t}}\left(a_{t}\right)\right)}^{s}\right)}^{s}}\\ \; & =\frac{\sum_{a_{t}}{\left(p_{j_{1}\ldots j_{t}}\left(a_{1},\ldots ,a_{t}\right)\right)}^{s}}{\sum_{a_{t}}{\left(p_{j_{t}}\left(a_{t}\right)\right)}^{s}}\;. \end{array}$$

After summing up in $a_{1},\ldots ,a_{t-1}$, we then have:

(107) $$\sum_{a_{1},\ldots ,a_{t-1}}{\left(\sum_{a_{t}}{\hat{p}}_{j_{t}}\left(a_{t}\right)p_{j_{1}\ldots j_{t}}\left(a_{1},\ldots ,a_{t}\right)\right)}^{s}\geq \frac{\sum_{a_{1},\ldots ,a_{t}}{\left(p_{j_{1}\ldots j_{t}}\left(a_{1},\ldots ,a_{t}\right)\right)}^{s}}{\sum_{a_{t}}{\left(p_{j_{t}}\left(a_{t}\right)\right)}^{s}}\;.$$

From the application of Bayes’ law to the right-hand side of Equation (107), we get Equation (47) again, i.e., the correspondence of the assumption of concavity has been derived once more. All the subsequent development of Section 4 will then follows accordingly.

## 7. Concluding Remarks

It should be stressed that the introduction of escort probabilities has been efficient on the construction of generalized entropy measures. These can be used for the classification of databases in terms of their clustering as driven by their intrinsic synergy and the resulting formation of more complex structures like families and clans.

A fundamental aim would be the derivation of a dynamical theory which would be able to describe the process of formation of these structures. A theory based on the evolution of the entropy values on databases which we hope that could be realized by methods introduced by the exhaustive study of Fokker–Planck equations.

Some introductory results on this promising line of research have been already published [16] and a forthcoming publication of a comprehensive review will summarize all of them.

## Data Availability

Not applicable.

## Abbreviations

The following abbreviations are used in this manuscript:

GKS	Generalized Khinchin–Shannon
SM	Sharma–Mittal
